# Inter-individual differences in weekly training load in international-level handball: the effect of congested match schedules

**DOI:** 10.3389/fspor.2025.1605750

**Published:** 2026-01-21

**Authors:** Daniel Büchel, Michael Döring, Jochen Baumeister

**Affiliations:** Exercise Science & Neuroscience Unit, Department Exercise and Health, Faculty of Science, University of Paderborn, Paderborn, Germany

**Keywords:** workload, fatigue, substitutional training, individualization, performance

## Abstract

**Background:**

Effective training load (TL) management is crucial for optimizing performance, especially in elite team sports, where congested schedules (≥ two matches a week) are common and can lead to fatigue accumulation. Understanding inter-individual variations in workload is a key to tailor training strategies. This study aimed to analyze inter-individual differences in accumulated TL in elite handball players over a macrocycle of 6 months with special regards to congested schedules.

**Methods:**

Fifteen professional players from the first German Handball League were monitored between July and December 2023. External (ACCLOAD, Kinexon, Munich) and internal load measures (sRPE) were recorded across 24 matches and 67 training sessions, The weekly workload accumulated during matches and training was analyzed descriptively. ANOVAs were performed to analyze the impact of the factor “week congestion”.

**Results:**

Analysis revealed substantial inter-individual differences in accumulated load, with coefficients of variation (CV) ranging from .14 to .27 depending on the measure (ACCLOAD, sRPE). Higher variation was observed during matches than during training, particularly in congested weeks. While no significant effect of week congestion was found for absolute weekly load, inter-individual variation increased in congested weeks. Case comparisons between backcourt and wing players highlighted discrepancies in load accumulation due to differences in playing time and role.

**Conclusion:**

These findings underscore the importance of monitoring individual TLs to optimize player performance and minimize fatigue particularly in congested schedules. Coaches should consider compensatory training to address these discrepancies. Our research provides valuable insights into load accumulation in elite team handball, emphasizing the need for personalized approaches to balance performance demands and recovery.

## 1 Introduction

Team handball is an indoor team sport played in teams of seven. Players face complex physical demands due to the sport's intermittent nature, where actions like sprints, accelerations, jumps, throws, and blocks are common (1–3). Depending on the individual roles of the players and their positions on the court, individual activity profiles emerge based on external training load data. Typically, wings cover long distances, play many minutes and perform many sprints. In contrast, backcourts and pivots play fewer minutes, but experience higher relative acceleration loads per minutes and more frequently perform jumps and changes of direction (4–6).

To prepare individuals optimally for these competitive demands, coaches can benefit from the monitoring of training load (TL) throughout the competitive season (7). In addition to the continuous recording of the aforementioned external TL indicators during training and matches, such as the acceleration load (ACCLOAD) metric, subjective and internal outcomes like the session Rating of perceived exertion (sRPE) provide valuable insights into the volume and intensity of a player's TL over longer periods (3, 7). On the one hand, the continuous monitoring of external and internal TL allows for the identification of players exposed to extraordinarily high TL, such as starters in soccer (8) or key players in handball and basketball (9). Upon identification, tailored recovery interventions can be prescribed. On the other hand, players with low chronic TL, such as bench players or youngsters, can be identified (8, 9). For these players, coaches may incorporate compensatory training to fill the TL gap compared to their starting teammates and ensure their long-term development (10).

The effective management of TL for both recovery and compensation must be implemented during training sessions, since match demands are largely fixed. Therefore, the long-term management of TL is particularly challenging in international-level team sports, because the time interval between two matches (= microcycle length) varies due to additional international appearances within the week (11). Such weeks with two or more matches are referred to as “congested weeks” because the number of matches per week increases while the recovery or compensation time defined by the number of training sessions decreases (12, 13). Retrospective analyses in soccer observed that subjective outcomes such as the sRPE decrease during congested weeks, likely as a function of tapering (14). Furthermore, it has been reported that objective external TL indicators such as covered distances and high speed running decrease in congested weeks (15). Therefore, the transferability of periodization patterns observed in professional teams with regular schedules (one match per week) to international level teams with congested schedules seems limited (7), highlighting the need to study and describe the training regimes of top-level teams.

Another issue resulting from congested schedules is their potential impact on inter-individual variations in TL within teams. Because teams train less and less hard due to tapering (14) but play more, it can be hypothesized that inter-individual differences in weekly training load associated with playing status (9) increase during congested weeks. Measures such as the coefficient of variation (CV) can reveal insights into variation of TL exposure within a team (16), with a higher CV describing a greater heterogeneity. For instance, Martin-Garcia et al. (2018) reported CVs around 19% to 20% for weekly distance and workload metrics in elite soccer, while Clubb et al. (2022) reporter between-player variations < 10% (17, 18).

Since technological limitations restricted the automatic and objective monitoring of TL in indoors-sports, retrospective analysis on TL accumulation in handball are sparse. However, the rise of automated wearable technologies such as inertial-motion-units (IMUs) and local-positioning-systems (LPS) (3, 19) facilitate TL monitoring in indoor sports. In a recent study, Font et al. (2023) pointed out the differences in TL regarding i) playing position and ii) training schedule in a second-division Spanish male handball team with a regular schedule (= one match/ week) based on ACCLOAD and sRPE metrics. Their analysis reveals that players experience highest external and internal TL on the match day and relatively lower external and internal TL on the two days before the match. Similar findings were reported by Holm & Randers (2025), who monitored external and internal training load in a first division Danish handball team (20). Their data also revealed a decrease in EL and IL across the microcycle as the match approached, highlighting the importance of microcycle periodization in professional team handball. However, their retrospective analyses are also based on data from regular schedules. Accordingly, data on accumulation of TL in international level team handball is lacking. Since evidence from other team sports indicates that microcycle periodization can be disturbed by additional international appearances (11, 14), a specific description of TL accumulation in congested weeks is warranted.

To improve our understanding of TL accumulation in elite team handball, this study aimed to analyze the inter-individual variations in TL of an international top-level handball team. This study retrospectively analyzed the recorded TL data of a German Bundesliga team. First, inter-individual differences in accumulated TL were analyzed using descriptive statistics including means, standard deviations and the CV. To gain deeper insight into these inter-individual differences, we conducted position-specific descriptive case-comparisons of two players of the same position with different playing statuses. By selecting the two players with the highest and lowest TL within each position, we retrospectively screened the maximum inter-individual TL difference over an extended period. Finally, we examined the specific impact of congested schedules (≥ two matches per week) as characteristic of international team handball. Therefore, both i) the average weekly TL and ii) the average inter-individual variation in TL were investigated with regard to congested weeks.

## 2 Methods

### 2.1 Subjects

Fifteen male professional handball players (28.0 ± 3.4 years; 192.5 ± 5.9 cm; 98.8 ± 9.7 kg) competing in the first German handball league participated in this study. Out of the fifteen players, 11 competed for their national team and played matches in one national match break in the first week of November. The TL accumulated during this period was not assessed. For descriptive analysis, players were grouped according to their usual playing position during the season (4 Wings, 3 Pivots, 6 Backcourts and 2 goalkeepers). Goalkeepers were included for descriptive, but excluded from statistical analyses due to their distinct activity profiles on the court (21). This study was conducted in accordance with the Declaration of Helsinki. Data collection was part of standard team monitoring procedures and was fully anonymized. According to institutional policy for the analysis of fully anonymized data collected as part of standard team monitoring procedures, formal ethical approval and written informed consent were not required.

### 2.2 Design

For the present study, an observational retrospective study design was selected. In addition to the Bundesliga, the analyzed team also participated in the German national cup and an european club competition. Therefore, the team had to perform two or more matches per week in 10 weeks of the investigated period, with additional traveling requested between the matches. TL monitoring was conducted on a daily basis during training and competition, and for the present investigation, the first significant period of the season lasting 23 weeks (July to December 2023) were analyzed. The present investigation includes 1,340 observations from 24 competitive matches (*n* = 361 observations) and 67 handball practice sessions (*n* = 979 observations). Strength & conditioning sessions performed during the season were not considered for TL analysis. Further, compensatory training sessions were not included in the analysis. Compensatory training sessions were conducted occasionally and took place on matchday + 1 and consisted of 15–20 min of agility drills and resistance exercises. TL was not recorded in these sessions because the player IMU system was not available in the gym facility where the training sessions took place.

### 2.3 TL monitoring

During the training sessions and cup matches, all players wore an IMU sensor near the scapula (Kinexon Perform IMU, Munich, Germany) to quantify overall movement and acceleration at a sampling rate of 100 Hz. Such sensors have been shown to quantify team sport-specific movements validly (22). During the league matches, a more sophisticated LPS with an integrated IMU (Kinexon Perform LPS, Munich, Germany) was utilized due to a league-wide contract. As for the IMU system, the wearable unit was positioned at the upper back to quantify overall movement and acceleration at a sampling rate of 100 Hz.

The ACCLOAD was extracted for both training and matches as outcome of interest based on the IMU data (4). ACCLOAD quantifies the overall amount of tri-axial motion based on the differentials of accelerometer data in a single number, reported in arbitrary units (au). While Saal et al. (2023) reported average values of around 250 aus per player with∼20 min on-court, Büchel et al. (2024) reported values around 480 aus per player with > 50 min on-court. Such measures have been shown to provide reliable estimates of overall physical activity (23). This metric is comparable to other indoor TL metrics previously used in handball such as the Player Load (7). However, it should be considered that manufacturer-set hardware, filtering and processing of the data can reduce the direct comparability between different systems.

ACCLOAD was not corrected for effective playing time to obtain comparable data for training and match sessions. Warm-Ups and breaks were not captured during training sessions and cup matches, so that focusing on effective playing time would underestimate the true physical activity on match days. Therefore, the overall amount of activity captured from the beginning to the end of the recording sessions contributed to ACCLOAD. Acyclic actions like sprints, and contacts were not considered because these rely on positional data, typically derived from LPS (24, 25).

The sRPE was assessed using a customized mobile application on a tablet as an internal load outcome. After each session, players were asked to rate their subjectively perceived exertion on a scale from 1 to 10. Then, the individual rating was multiplied by the session duration in minutes, resulting in a final score in arbitrary units (au) (26). ACCLOAD and sRPE are sensitive to differences in TL in team handball related to different micro- and macrocycles (7, 27). As for ACCLOAD, effective playing time during matches was not considered because it underestimates the true activity including warm-up and halftime-activation. Session duration was set to 105 for all matches as default, since this time covers the estimated total duration of warm-up, absolute playing time and halftime activation.

### 2.4 Statistics

Data were analyzed and visualized using customized MATLAB scripts (Software Version R2023a). The inter-individual differences in seasonal load were analyzed descriptively. All available values (ACCLOAD_Overall_ and sRPE_Overall_) per player were summed up across the investigated period. We further specified external and internal load accumulation originating from training sessions (ACCLOAD_Training_; sRPE_Training_) and load originating from competitive matches (ACCLOAD_Match_; sRPE_Match_). For the case comparison, two back and wing players with substantial ACCLOAD discrepancies were selected. Using the week-to-week differentials of the accumulated load of the two players, the continuous accumulations of TL differences over the season were visualized.

We also investigated changes in weekly TL in relationship to the factor “week congestion”. Since tapering towards the matchday has been observed in two independent studies in professional handball (7, 20), weeks were categorized depending on the number of matchdays, resulting in weeks with no match (*n* = 5), normal weeks with one match (*n* = 8) and congested weeks with two or more matches (*n* = 10). For practicability, weeks were considered to start on Mondays and end on Sundays To investigate the influence of congested schedules on the absolute weekly ACCLOAD and sRPE, we used a linear mixed-effects model, fitted using the fitlme function in MATLAB (R2023a). The model included ACCLOAD/ sRPE as the dependent variable and Week Type (“Normal”, “Congested”, “No Match”) as the independent variable. To account for non-independence of repeated measurements from the same individual, Player was included as a random effect, with a random intercept estimated for each of the 13 field players. Congested Weeks were set as reference level for the Week Type factor. The model was fitted using maximum likelihood (ML) estimation. To quantify the size of the significant differences, we calculated Cohen's *d*-like effect size and interpreted them as small (≥.2), medium (≥.5) and large (≥.8).

For the comparison of CVs across normal weeks, congested weeks and weeks with no match, Kruskal–Wallis tests were performed, since Shapiro–Wilk tests revealed that data was not normally distributed (*p* > .05). CV was calculated as the ratio between the weekly standard deviation and the weekly mean accumulated load. For the detection of *post hoc* differences, the Tukey-Kramer correction was applied (*p* < .05). Effect sizes for main effects were derived from eta-squared based on the H statistic (*η*H2) and interpreted as: small (>.01), medium (>.06) and large effects (.014).

## 3 Results

### 3.1 Inter-individual differences in accumulated load

**Figure 1:**
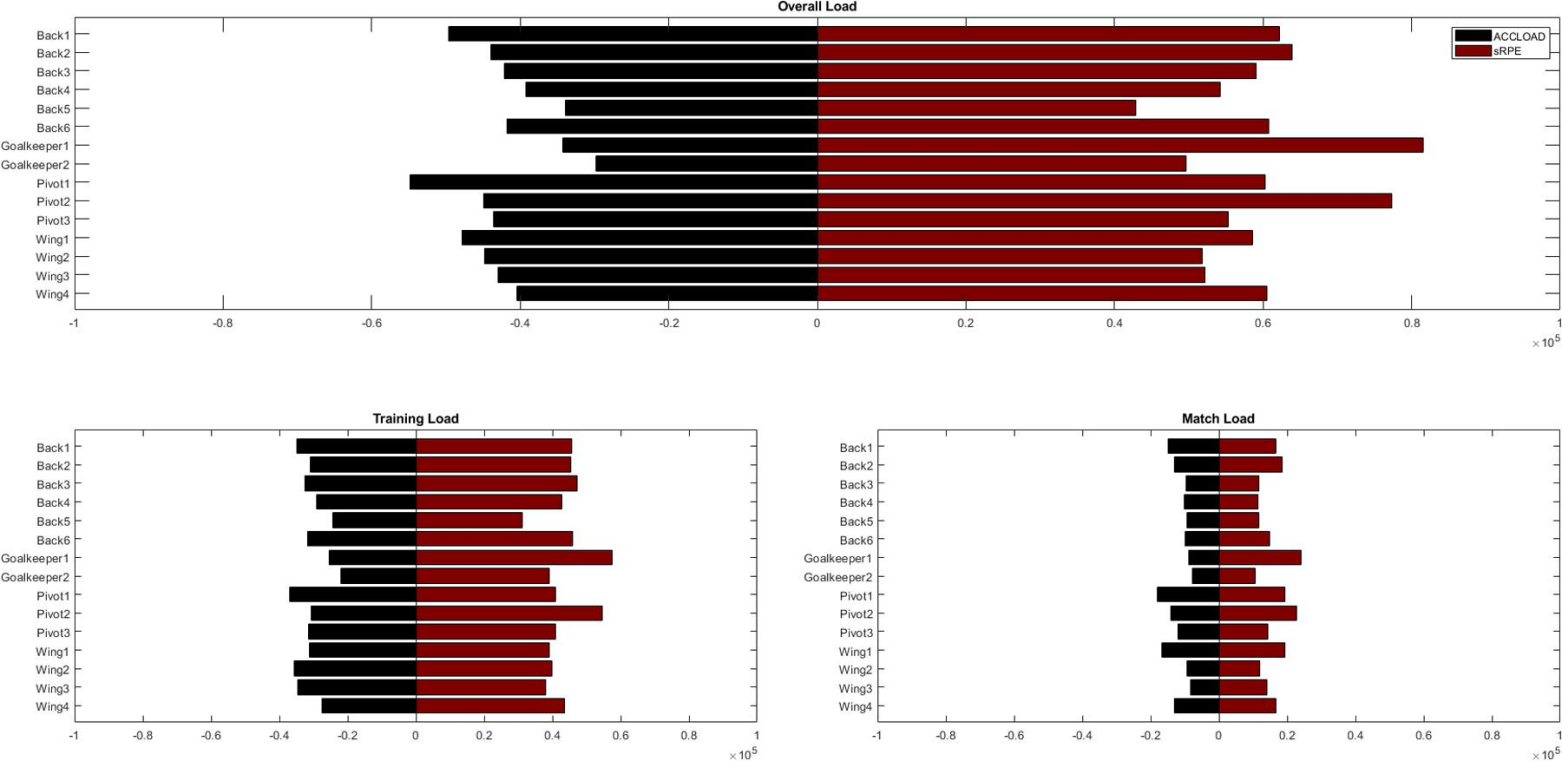
Inter-individual differences in accumulated acceleration load (ACCLOAD, black bars) and session rating of perceived exertion (sRPE, red bars) in an international-level handball team. Top tile visualizes overall load accumulated across the season, bottom left tile visualizes load accumulated during training sessions, bottom right tile indicates load accumulated during matches for each player.

Descriptive analyses of accumulated seasonal load revealed inter-individual differences in accumulated TL within the observed team. A visualization of all metrics with regard to the individual players sorted by position can be found in Figure 1.

For the ACCLOAD_Overall_ and sRPE_Overall_, CVs of .15 and .16 have been observed, respectively. For ACCLOAD_Match_ and sRPE_Match_, increased CVs around .27 have been observed, while CVs for ACCLOAD_Training_ and sRPE_Training_ were .14 and .15, respectively. An overview of means, SDs and CVs are reported in Table 1.

**Table 1:** Mean accumulated load throughout 23 weeks in elite male team handball.

Training load variable	Mean ± SD	CV
ACCLOAD_Overall_	42,277.84 ± 6,373.95	0.15
ACCLOAD_Training_	30,595.72 ± 4,285.53	0.14
ACCLOAD_Match_	11,682.12 ± 3,155.59	0.27
sRPE_Overall_	56,844.00 ± 9,287.07	0.16
sRPE_Training_	43,204.00 ± 6,431.97	0.15
sRPE_Match_	13,640.00 ± 3,639.29	0.27
ACCLOAD_Match/Training ratio_	0.32 ± 0.09	0.27
sRPE_Match/Training ratio_	0.24 ± 0.06	0.27

Accumulated acceleration load (ACCLOAD) and session rating of perceived exertion (sRPE) are provided as overall values and training- and match-specific values, respectively.

### 3.2 Case-comparisons of TL accumulation

Apart from a descriptive analysis of the complete team, we also performed case comparisons of two specific players of the same playing position with different playing states. Both analyzed backcourts performed as central/ left backs and participated a comparable number of training sessions and competitive matches during the period of interest. While Back 1 accumulated the highest ACCLOAD_Overall_ of all backcourts, Back 4 served as a substitute and entered the game for limited periods. Both players were chosen since they were spared from injuries during the investigation period. A descriptive overview of both backcourtś exposure including percentage differences is shown in Table 2.

**Table 2:** Description of seasonal exposure of the two backcourt players included in the case comparison.

Training load variable	Back 1		Back 4	
Training sessions (*n*)	66		63	
Duration (min)	90.3	(19.7)	91.13	(20.8)
ACCLOAD_Training_ (min)	528.2	(140.5)	460.3	(131.9)
sRPE_Training_ (au)	538.8	(193.0)	641.5	(187.0)
Matches (*n*)	31		30	
Duration (min)	96.9	(17.4)	97.7	(18.1)
ACCLOAD_Match_ (min)	478.3	(61.0)	341.1	(131.9)
sRPE_Match_ (au)	561.5	(269.6)	599.2	(285.4)

While Back 1 was the team's starter, Back 4 served as a substitute and entered the game for limited periods. Durations are displayed as total times, including breaks and time on the bench.

**Figure 2:**
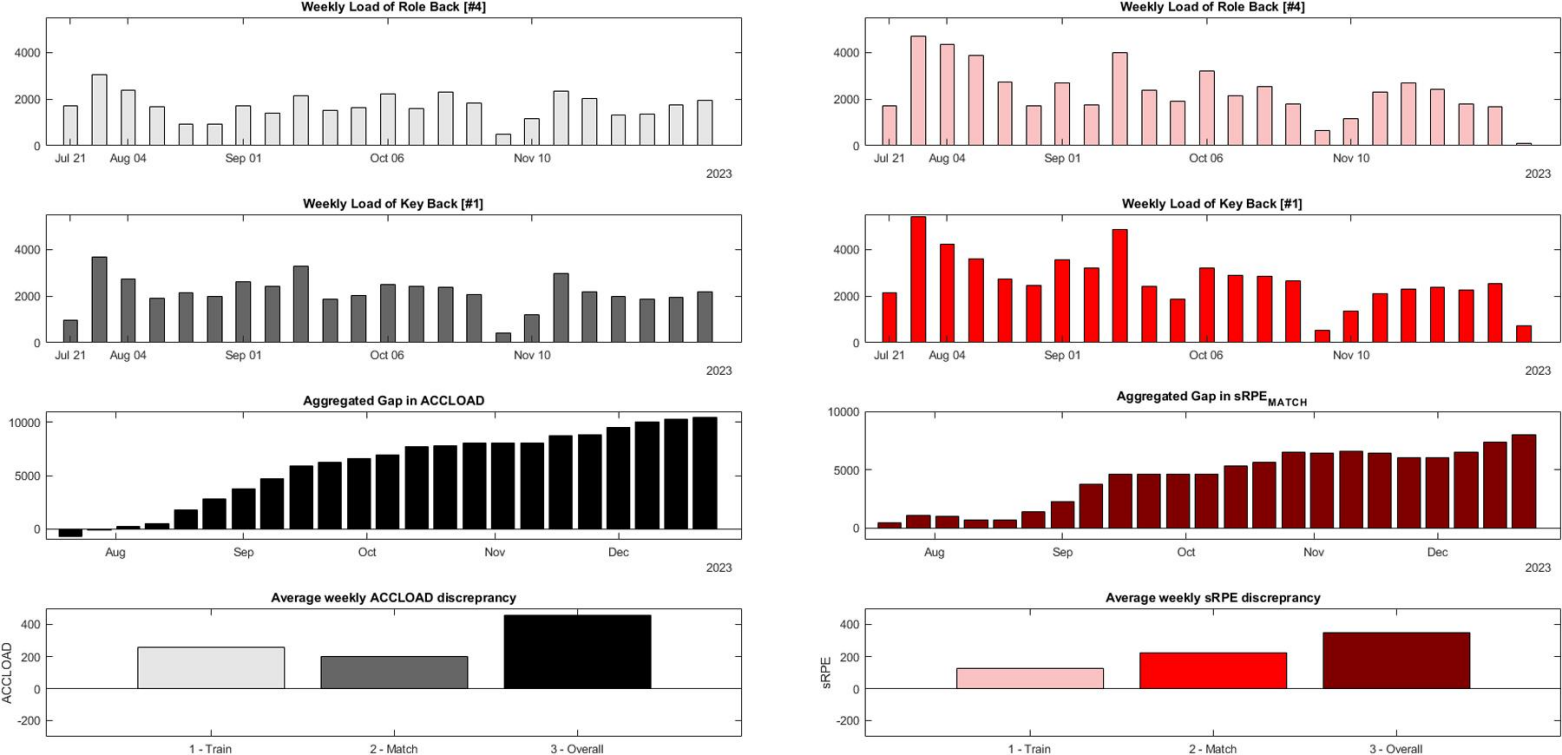
Case-comparisons between two backs regarding the inter-individual differences in i) accumulated weekly acceleration load (black bars) and session RPE (red bars) in the first round of the season 2023/2024. Back 1 was a starter, while Back 4 was a substitute with reduced playing time. The left half of the chart depict accumulated acceleration load (ACCLOAD) as an objective index of external load. In contrast, the right half of the chart depicts session rating of perceived exertion (sRPE) as a subjective index of internal load. Each bar reflects the accumulated TL of a week starting on Monday and ending on Sunday. The first row displays the weekly load of Back 4, and the second row displays the weekly load of Back 1. The third row shows the accumulation of load discrepancy across the season concerning overall load. The bottom row displays the mean weekly discrepancy for training, match, and overall load.

Figure 2 lines out the continuous accumulation of load discrepancy between both players. On average, Back 1 accumulated around ∼70 more aus of ACCLOAD and ∼170 more aus of sRPE per training and ∼140 more aus of ACCLOAD and ∼40 more aus of sRPE per match.

After the period of interest, the inter-individual discrepancy in ACCLOAD_Overall_ counted∼11,100 au and 6,400 au in sRPE_Overall_, respectively. An overview of the temporal accumulation of differences in ACCLOAD and sRPE can be found in Figure 2.

Further, we compared inter-individual differences in accumulated TL in two wings. Wing 1 performed as left wing, Wing 2 performed as right wing, and both participated in 62 training sessions and 31 competitive matches during the period of interest. While Wing 1 accumulated the highest ACCLOAD_Overall_ of all wings and was a starter, Wing 2 served as a substitute and entered the games for short periods. Both players were spared from injuries during the investigation period. A descriptive overview of both player's exposure including percentage differences is shown in Table 3.

**Table 3:** Description of the seasonal exposure of the two wing players’ included in the case comparison.

	Wing 1		Wing 2	
Training Sessions (n)	**62**		**62**	
Duration (min)	91.19	(20.5)	90.7	(21.0)
ACCLOAD (min)	502.1	(131.2)	573.5	(149.5)
sRPE (au)	714.8	(381.5)	872.3	(177.6)
				
Matches (n)	**31**		**31**	
Duration (min)	97.8	(17.7)	96.9	(18.2)
ACCLOAD (min)	455.0	(95.6)	299.9	(207.3)
sRPE (au)	515.7	(258.9)	523.2	(250.3)

While Wing 1 was the team*’*s starter, Wing 2 served as a substitute and entered the game for short periods. Durations are displayed as total times, including breaks and time on the bench.

**Figure 3:**
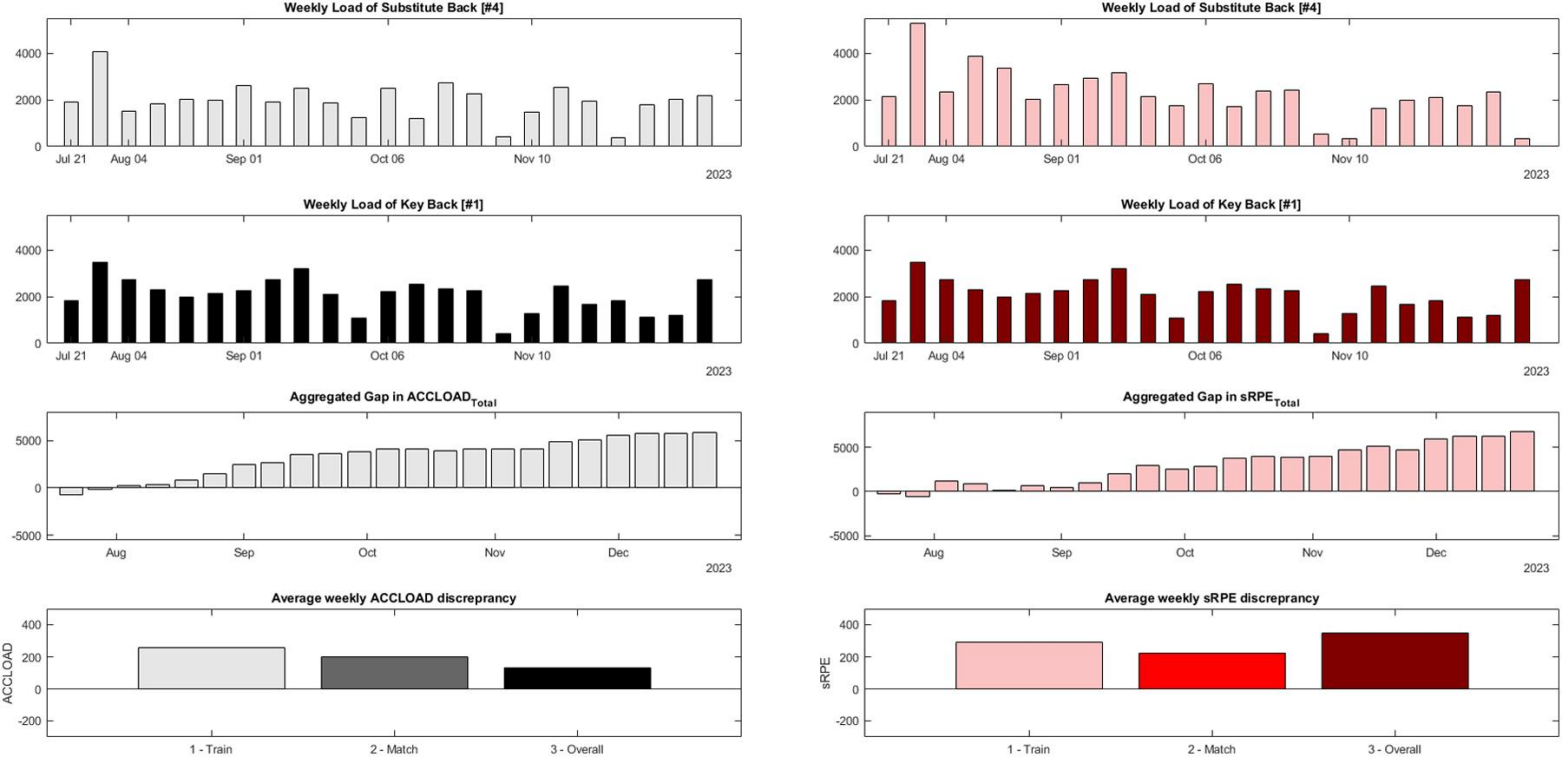
Case-comparisons between two wings regarding the inter-individual differences in i) accumulated weekly acceleration load (black bars) and session RPE (red bars) in the first round of the season 2023/2024. Wing 1 was a starter, while Wing 2 was a substitute with limited playing time. The left half of the chart depict accumulated acceleration load (ACCLOAD) as an objective index of external load, while the right half of the chart depicts session rating of perceived exertion (sRPE) as a subjective index of internal load. Each bar reflects the accumulated TL of a week starting on Monday and ending on Sunday. The first row displays the weekly load of Wing 2, and the second row displays the weekly load of Wing 1. The third row shows the accumulation of load discrepancy across the season concerning overall load. The bottom row displays the mean weekly discrepancy for training, match, and overall load.

On average, Wing 1 accumulated ∼131 more aus of ACCLOAD and ∼293 more aus of sRPE per session. In contrast, Wing 2 accumulated more ACCLOAD and sRPE per training (∼192 and ∼25 aus, respectively), while Wing 1 accumulated more ACCLOAD and sRPE per match (∼323 and ∼318 aus, respectively). After the period of interest, the inter-individual discrepancy in ACCLOAD_Overall_ counted∼3,000 aus and 6,700 aus in sRPE_Overall_, respectively. Figure 3 displays the continuous accumulation of load discrepancy between both wings.

### 3.3 Absolute weekly TL in congested weeks

The fixed effect of Week Type did not show a statistically significant overall effect on ACCLOAD [*F*(_2,285_) = 1.2471, *p* = .289]. The variance explained by the random effect of Player was negligible (SD < 0.01).

Examining the model's fixed-effect coefficients, with “Congested” weeks as the reference, ACCLOAD during “No Match” weeks was not significantly different (*b* = 81.90, 95% CI [−127.32, 291.13], t(**_285_**) = 0.77, *p* = .442). Likewise, ACCLOAD during “Normal” weeks was not significantly different from “Congested” weeks (*b* = 144.55, 95% CI [−36.71, 325.81], *t*(**_285_**) = 1.57, *p* = .118).

The fixed effect of Week Type did not show a statistically significant overall effect on sRPE [*F*(_2,285_) = 2.07, *p* = .129]. The variance explained by the random effect of Player was negligible (SD < 0.01).

When examining the specific model coefficients (with “Congested” weeks as the reference), there was no difference in sRPE during “No Match” weeks (*b* = 18.90, 95% CI [−328.83, 366.62], *t*(_285_) = 0.11, *p* = .915). There was a non-significant trend suggesting that sRPE during “Normal” weeks were higher than during “Congested” weeks, but this effect did not reach the threshold for statistical significance (*b* = 293.79, 95% CI [−9.16, 596.73], *t*(_285_) = 1.91, *p* = .057).

**Figure 4:**
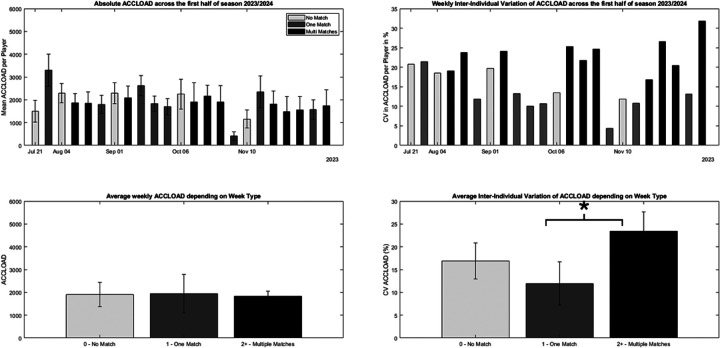
Schematic overview of accumulated weekly acceleration load (left side) and inter-individual variation in weekly acceleration load (right side) in a group of 13 male international level handball players. Light grey bars indicate weeks with no match (*n* = 5), dark grey those with one match (*n* = 8) and black bars those with two or more matches per week (*n* = 10). The top tiles display the weekly mean load and inter-individual variability in weekly load across the period of interest for ACCLOAD, respectively. Bottom tiles display the grand means and SDs for the different week types.

**Figure 5:**
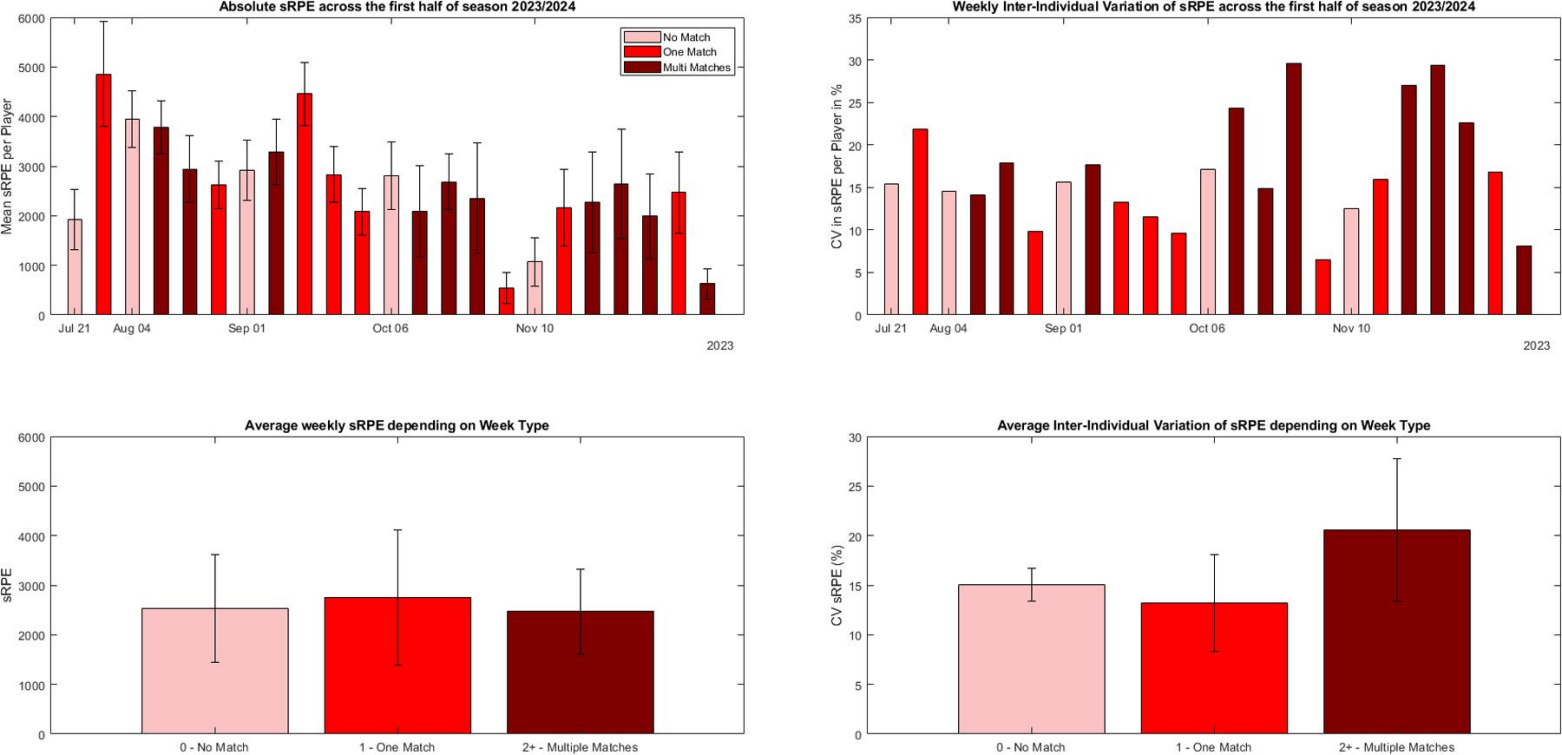
Schematic overview of accumulated weekly session RPE (left side) and inter-individual variation in weekly sRPE (right side) in a group of 13 male international level handball players. Light red bars indicate weeks with no match (*n* = 5), mid red those with one match (*n* = 8) and deep red bars those with two or more matches per week (*n* = 10). The top tiles display the weekly mean load and inter-individual variability in weekly load across the period of interest for sRPE, respectively. Bottom tiles display the grand means and SDs for the different week types.

An overview of absolute ACCLOAD and sRPE across week types can be found in Figures 4, 5, respectively.

### 3.4 Variability of weekly TL in congested weeks

A Kruskal–Wallis H test showed that there was a significant effect of Week Type on the ACCLOAD [*H*(_2_) = 13.77, *p* = .001], associated with a large effect size of *η*^2^*_H_* = .63. For sRPE, a non-significant effect of Week Type was observed [*H*(_2_) = 5.96, *p* = .51]. *Post-hoc* analyses revealed significantly higher variations for matches with multiple matches ACCLOAD (*p* = .001) for weeks with more than one matches compared to weeks with one match. An overview of the weekly inter-individual variation of ACCLOAD and sRPE can be found in Figure 4, 5, respectively.

## 4 Discussion

The current investigation aimed to quantify the inter-individual differences in TIL observed in an elite handball team across the first half of a competitive season. Our findings demonstrated that seasonal TL showed substantial variations across players for both external and internal load indicators. These variations were evident across the whole team and became even more pronounced when analyzing individual case comparisons. In addition, our statistical analyses revealed that the weekly number of matches moderates the inter-individual variation of TL, with higher variations observed in congested weeks. Together, the current study's findings provide novel data on TL monitoring in elite-level team sports and offer realistic insights into the challenges faced by coaches and scientists.

### 4.1 Substantial inter-individual differences in accumulated TL

The descriptive analysis of accumulated TL revealed substantial differences within the analyzed team. The computation of CoV revealed inter-individual variations of 16%–27% of TL, depending on the outcome investigated. The most extensive variation was observed for load accumulated during competitive matches, which was 27% and 26% for ACCLOAD and sRPE, respectively. These values are comparable to data reported by Martin-Garcia (2018) on inter-individual variability of weekly external TL in international level soccer players (16). Therefore, balancing TL can be considered as a key challenge in elite team sports.

In total, the standard deviation of accumulated TL throughout 23 weeks of a competitive season was∼6,300 au of ACCLOAD and 9,300 au of sRPE. Considering previous analyses using the same system in official Bundesliga matches, players with substantial playing time accumulate around 500 au of ACCLOAD per match (4). Therefore, the observed standard deviation in TL resembles the equivalent of around 13 full Bundesliga matches within only 23 weeks of a competitive season. Competitive matches serve as an important physiological stimulus in team sports (7, 28) and expose individuals to unique technical and tactical demands. Since Silva et al. (2011) observed that the development of athletic performance throughout a competitive season was associated with playing status, we may speculate that substitute players miss important stimuli for adaptation and development and need compensatory training players (29). Further, the imbalance in TL exposure may also lead to a significant accumulation of fatigue, since ACCLOAD metrics have been strongly associated with the individual perception of fatigue (30). Here, Cruz et al. (2018) observed that increased TL accumulation may lead to short-term reductions in jumping performance as a function of fatigue (31). Therefore, monitoring external load over more extended periods seems important to identify under- and overexposure within professional teams. Due to the limited research on in-season measures related to compensatory training and recovery in team sports, we underscore the need to monitor such approaches in professional sports. For instance, sports scientists could split teams according to accumulated TL and monitor whether players’ performance changes over time (10).

Our case comparisons support the observation of vast TL gaps on an individual level. In both comparisons, the different playing playing status resulted in substantial differences in TL after the investigated period. Two interesting observations can be highlighted. In the backs, the gap in TL originated not only from differences in ACCLOAD_Match_ but also from ACCLOAD_Train_. Therefore, the pivotal role of key players seems to be also expressed through increased load accumulation during training sessions since key players may run more attacking and defending formations and trials during training. A report from the NBA, where starters were exposed to more weekly TL despite having less absolute on-court training time, is in line with these observations (32). For the two wings compared, the accumulation of TL across training and matches showed different patterns compared to the pair of backcourts. While Wing 2 outperformed Wing 1 regarding ACCLOAD_Training_, Wing 1 outperformed his counterpart regarding ACCLOAD_Match_. It remains unclear why the wing role player outperformed his key player counterpart, while the backcourt role player did not. It could be speculated that various aspects such as the pre-training recovery state (33), defensive/ offensive specialization (34) or the fitness level (35) modulate these individual patterns. Taken together, the observations on persistent differences in TL between starters and rotation players are a key benefit of systematic TL monitoring. For transfer into TL monitoring routines, coaches should further consider that activity profiles vary among playing positions (Büchel et al., 2024, Font et al. 2021). While the ACCLOAD is a generic outcome which describes overall activity of the player on the court, coaches should also integrate TL markers such as jumps, sprints, or collisions to fill the gap between key and role players as specifically as possible.

### 4.2 Congested weeks increase inter-individual variation of TL

Our analyses revealed that absolute TL did not increase in congested weeks. This null finding may support the tapering hypothesis mentioned in the introduction. It is plausible that in congested weeks, the reduced load from fewer or less intense training sessions is offset by the increased load from additional matches, resulting in a comparable total weekly load across the team. However, the composition of that load (i.e., match vs. training) appears drastically different in congested weeks and likely drives the increased inter-individual variation observed. Interestingly, retrospective analyses in international level soccer revealed that the number of matches per week modulates periodization and leads to reduced average TLs in the training sessions close to the match to taper performance (11, 15, 36). Due to the limited and variable number of training sessions in the congested weeks, a specific analysis of microcycle periodization as performed by Font et al. (2022) or Holm & Randers (2025) based on the actual schedule of the analyzed team. Hence, it remains speculative whether periodization changes in international level handball players during congested schedules.

A critical point of divergence and novelty compared to previous studies by Font et al. (2023) and Holm & Randers (2025) is that these studies reported TL based on regular schedules. Their work established clear tapering towards the one match day of the week. Our report suggests that these established patterns are disturbed or replaced during congested weeks. Since players only train once to twice between matches in congested weeks tapering opportunities are limited. Therefore, our data extends the existing literature on TL monitoring in team handball by showing that schedule congestion *per se* does not amplify total load.

While congestion did not amplify absolute TL, the congestion of the schedule served as a moderator of the inter-individual differences in TL, as the mean CV of weekly load increased in congested weeks. In line with existing research, it might be speculated that the tapering in congested weeks plays a role here (11, 15, 36). Since coaches reduce training intensity in congested weeks to promote the recovery of key players, role players do not reach sufficient training stimuli in these periods. Furthermore, additional travel may interfere with recovery and force coaches to taper training intensity (37). Thus, differences in match load arising from match status are emphasized and cannot be compensated due by additional training (4).

Accordingly, congested periods seem particularly important in training monitoring, since the risk of over- and underloading appears to be higher. To optimize compensatory training and fatigue prevention, coaches may anticipate the increased inter-individual variation in such weeks and intervene with tailored training programs designed for substitutes and key players. Since congested weeks are likely to expose team sports athletes to higher risks of injury, the overloading of key players should be minimized (13). In this context, the CV may serve as a suitable parameter to identify periods where TL gaps within a team increase. For instance, CV values for weekly accumulated TL > 20% could be used as benchmarks to flag unbalanced weeks and prescribe additional training sessions for underloaded players. Such sessions should include bursts of high-intensity and multidirectional drills to expose low-TL players to handball-specific neuromuscular training stimuli (Büchel et al. 2024). As one of the first studies investigating different types of compensatory training sessions, Diaz-Serradilla et al. (2023) pointed out that a combination of small-sided games and running-based drills is most effective to simulate the match demands for role players. Particularly in advance of congested weeks, coaches can schedule such training sessions for role players to anticipate and prevent gaps in training load accumulating from more matches, more traveling and fewer training sessions. Scheduling sessions on the matchday itself or the day after are stated to be beneficial because recovery processes between key and role players remain synchronized (Casamichana et al., 2024).

### 4.3 Limitations

The present study is not without limitations. First, the sample utilized is limited to a single team monitored for only half of a competitive season. Therefore, the influence of a specific coaching philosophy and player rotation strategy must be considered (38). For more generalizable insights, future research should include multiple teams with different philosophies. Furthermore, comparisons to control teams with normal schedule would be important to better understand the impact of schedule congestion. While our data was monitored automatically during competition and training, reflecting real-world practices to a high degree, the single-team design limits the external validity of our findings.

From a technical perspective, our methodology has several constraints. Freitas et al. (2025) demonstrated that linear player load metrics are likely to overestimate true activity in presence of short bouts of high-intensity acceleration such as collisions. Therefore, the ACCLOAD values of players exposed to frequent collisions are likely overestimated in our study. Adapted algorithms such as the Body Load metric can help to remove these components from the final TL metric through a combined linear and cubic interpretation of the raw data (Freitas et al, 2025). Additionally, Dawson et al. (2024) claim that accumulated acceleration load metrics lack consideration of temporal load patterns, acceleration variability, and the number and magnitude of individual load periods. Therefore, future research is needed to also consider temporal aspects like peak intensity periods into load training monitoring (39). At the same time, metrics on peak intensity could serve as valuable benchmarks for compensatory training sessions, since they allow to prepare players for the most demanding passages of the match. For instance, training drills can be designed which simulate the three most intense minutes of a handball match.

Our analysis was also limited by the type of data collected. Acyclic activities like jumps and sprints were not included in the analyses, since we only had access to an IMU-based system during training. Monitoring external TL through other technologies like LPS systems could also provide valuable insights into TL accumulation and serve as benchmarks for compensatory training design (4). Consequently, the integration of different TL monitoring techniques, such as video, IMU, and LPS, is recommended to quantify TL and the resulting inter-individual differences as holistically as possible.

Furthermore, it is important to note that compensatory training sessions were not included in the analysis of the present study. Although such sessions were not performed after every single match, their omission means the activities performed are missing from this report. Accordingly, the true TL of role players was likely underestimated while the gap between key and role players was likely overestimated. Nevertheless, the large gap observed states a developmental concern and the observed inter-individual differences in TL from team training and matches remain. Since compensatory training aims to close this gap, it becomes even more important to monitor compensatory training sessions to understand their composition and quality. Therefore, future studies should focus on compensatory training and investigate whether such sessions can effectively bridge TL gaps.

As a final limitation, it must be admitted that this study lacks data on cause-effect relationships between TL discrepancies and performance changes are lacking. The present study describes a data-based observation on inter-individual variations in elite handball, but the consequences of this gap on performance development remain speculative. Therefore, future studies must track TL and neuromuscular performance over time to understand whether under- or overloading players over time actually has a negative effect on physical performance. In their meta-analysis, Claudino et al. (2017) report that regular assessments of Counter Movement Jump height provide valuable insights into performance development and fatigue. Thus, it is important to address associations between TL and changes in performance in upcoming research.

### 4.4 Practical application & future works

Our insights from elite team handball aim to draw coaches' and scientists' attention to the risk of over- and underexposing individual players.

Therefore, our first recommendation is to quantify inter-individual differences in weekly training load (TL) using variability measures such as the coefficient of variation (CV). Defining arbitrary thresholds (e.g., a CV ≥ 20%) can help flag weeks with an increased risk of individual under- or overexposure. In such weeks, coaches should consider additional training for underloaded players while implementing effective recovery strategies for overloaded players. For instance, additional travel during congested weeks can interfere with recovery; interventions focusing on sleep hygiene and nutrition can help counteract travel fatigue (van Rensburg et al., 2021).

Our second recommendation is to include both external and internal TL metrics to identify inter-individual differences in TL accumulation. Our comparison of ACCLOAD and sRPE indicates that combining measures of accumulated physical workload and perceived workload can reveal unique insights into short- and long-term imbalances in TL exposure. The sRPE is a psychophysiological measure affected not only by physical factors but also by emotional and psychological ones. For instance, Kong et al. (2024) revealed that issues such as sleep deprivation can exacerbate perceived exertion. Therefore, including cognitive and psychological factors, in addition to physical ones, is crucial for identifying players at acute risk of overload. Taken together, implementing variability coefficients into coaching dashboards can support coaches in anticipating and identifying TL imbalances, such as those we observed during congested weeks.

While this study provides an initial insight into TL management in elite sports, future work is imperative to understand the short-, mid-, and long-term consequences of imbalances in accumulated TL. Most importantly, future work should aim to establish links between TL variability and objective performance outcomes. Investigating the association between under- and overloading players and changes in outcomes like jumping or sprinting performance is needed to validate the inclusion of TL variability benchmarks in monitoring routines.

Furthermore, future studies with larger samples from multiple teams should expand our generic approach to a more position-specific analysis of TL variability. Such an approach would allow for capturing the under- and overexposure of players relative to their distinct activity patterns and key performance indicators, such as collisions for pivots, changes of direction for backcourt players, and sprints for wings. Finally, future studies should evaluate suitable thresholds for interpreting the CV of inter-individual TL variability. While fixed thresholds could help identify risks in a standardized way, machine-learning approaches may be more suitable for predicting deviations in accumulated TL within teams.

## 5 Conclusion

The present study is the first one to provide insights into the inter-individual variation in TL in international level team handball. We observed considerable variations in accumulated TL on the group and case level. The implementation of compensatory and recovery strategies might help to close these gaps. Additionally, we observed that inter-individual variations are particularly present in congested weeks. Coaches should consider this phenomenon through careful and inter-individual adaptations of training in these periods.

## Data Availability

The raw data supporting the conclusions of this article will be made available by the authors, without undue reservation.
